# Revascularization in frail patients with acute coronary syndromes: a retrospective longitudinal study

**DOI:** 10.1093/eurheartj/ehae755

**Published:** 2024-11-16

**Authors:** Marius Roman, Joanne Miksza, Florence Yuk-Lin Lai, Shirley Sze, Katrina Poppe, Rob Doughty, Iain Squire, Gavin James Murphy

**Affiliations:** Department of Cardiovascular Sciences and National Institute for Health Research Leicester Biomedical Research Unit in Cardiovascular Medicine, University of Leicester, Groby Road, Leicester LE3 9QP, UK; Department of Cardiovascular Sciences and National Institute for Health Research Leicester Biomedical Research Unit in Cardiovascular Medicine, University of Leicester, Groby Road, Leicester LE3 9QP, UK; Department of Cardiovascular Sciences and National Institute for Health Research Leicester Biomedical Research Unit in Cardiovascular Medicine, University of Leicester, Groby Road, Leicester LE3 9QP, UK; Department of Cardiovascular Sciences and National Institute for Health Research Leicester Biomedical Research Unit in Cardiovascular Medicine, University of Leicester, Groby Road, Leicester LE3 9QP, UK; Section of Epidemiology and Biostatistics, School of Population Health, University of Auckland, Auckland, New Zealand; Department of Medicine, Faculty of Medical and Health Sciences, University of Auckland, Auckland, New Zealand; Greenlane Cardiovascular Service, Auckland District Health Board, Auckland, New Zealand; Department of Medicine, Faculty of Medical and Health Sciences, University of Auckland, Auckland, New Zealand; Greenlane Cardiovascular Service, Auckland District Health Board, Auckland, New Zealand; Department of Cardiovascular Sciences and National Institute for Health Research Leicester Biomedical Research Unit in Cardiovascular Medicine, University of Leicester, Groby Road, Leicester LE3 9QP, UK; Department of Cardiovascular Sciences and National Institute for Health Research Leicester Biomedical Research Unit in Cardiovascular Medicine, University of Leicester, Groby Road, Leicester LE3 9QP, UK

**Keywords:** Frailty, Acute coronary syndrome, Hospital episode statistics, Cardiovascular outcomes, Impact

## Abstract

**Background and Aims:**

Frailty is increasingly prevalent in people presenting with acute coronary syndrome (ACS). This high-risk group is typically excluded from trials of interventions in ACS, and there is uncertainty about the risks and benefits of invasive management.

**Methods:**

Patients with an ACS diagnosis between 2010 and 2015 in England were identified from Hospital Episode Statistics, with linked Office for National Statistics mortality data. Frailty was defined by the Hospital Frailty Risk Score. Causal inference analysis used regional variation in revascularization as an instrumental variable to estimate average treatment effects of revascularization on cardiovascular mortality up to 5 years in people presenting with ACS and low-, intermediate-, or high-risk frailty.

**Results:**

The analysis included 565 378 ACS patients, of whom 11.6% (*n* = 65 522) were at intermediate risk and 4.7% (*n* = 26 504) were at high risk of frailty. Intermediate and high frailty risks were associated with reduced likelihood of echocardiography, invasive angiography, or revascularization and increased likelihood of mortality and major adverse cardiovascular events compared with low frailty risk. Cardiovascular death at 5 years was 78.6%, 77.3%, and 75.7% in people at low, intermediate, and high frailty risk, respectively. Instrumental variable analysis suggested that revascularization resulted in a higher absolute reduction in cardiovascular mortality in high and intermediate frail risk patients compared with low risk at 1-year post-ACS.

**Conclusions:**

Frailty is common in people presenting with ACS, where cardiovascular causes are the principal mode of death. Revascularization is associated with short- and long-term survival benefits in people at intermediate and high risk of frailty after adjustment for measured and unmeasured confounders.

## Structured Graphical Abstract

**Structured Graphical Abstract:**
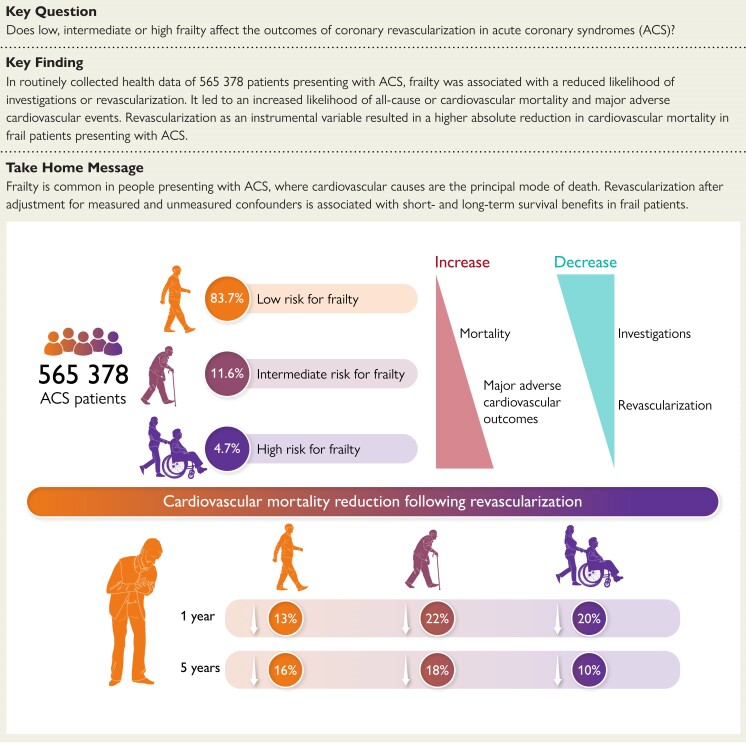
Graphical representation of the total number of acute coronary syndrome (ACS) patients and proportion of patients at low risk, intermediate risk, and high risk for frailty. Higher frailty risk led to an increase in mortality and major adverse cardiovascular events, with less likelihood of being investigated or receiving revascularization. Revascularization reduced cardiovascular mortality at 1 and 5 years, with a higher reduction at 1 year in the intermediate- and high-risk frailty groups.

**See the editorial comment for this article ‘Handle with care, proceed with caution: do frail patients presenting with acute coronary syndrome benefit from revascularization?’, by A. Oliva and R. Mehran, https://doi.org/10.1093/eurheartj/ehae623.**

## Introduction

Improving the lives of people with frailty and cardiovascular disease is a priority for clinical research.^1–3^ With an ageing population, an increasing number of people presenting with acute coronary syndrome (ACS) are frail. Frailty is associated with poor clinical outcomes.^4–6^ However, evidence to guide treatment decisions in this high-risk group is limited as multiple comorbidities, dementia, or frailty are common exclusions for randomized controlled trials (RCTs) of interventions in ACS.^7^ The 2023 European Society of Cardiology guidelines for managing ACSs highlight the increased risk in people with frailty, the need for individualised holistic treatments, and limited RCT evidence to inform management.^8^ Recruiting people with frailty to RCTs is challenging, and trial populations consistently include fewer elderly, frail, or people with multiple long-term conditions than the target population for the intervention.^7^

While awaiting evidence from high-quality RCTs in this population, modern causal inference methods can be used to inform treatment decisions.^9,10^ These methods overcome many common biases in observational analyses that arise from unmeasured confounders, such as reverse causation and bias by indication. A common feature of low certainty evidence to guide clinical decisions is wide variation in treatment independent of the individual patient’s characteristics and clinical outcomes.^11^ This random variation can be used as a statistical instrument to infer relationships between treatment choice and outcomes independently of measured and unmeasured confounders.^12^ The current study aims to use national inpatient healthcare records in England to determine the variation of coronary revascularization in people at low, intermediate, and high risk of frailty who present with ACS across geographical regions in England. Then, using causal inference analyses, we will determine the association between revascularization and important short- and long-term clinical outcomes. The results provide a rationale for future trials in ACS patients with a high risk for frailty and point towards other important unmeasured considerations in decision-making in these cohorts.

## Methods

### Design

A cohort of patients with ICD-10 codes^13^ used to define ACS was identified in the Hospital Episode Statistics (HES) for England between 2010 and 2015. All information in HES on these patients was collected 2 years before their index date to define frailty and comorbidities. Five years of follow-up was used to measure outcomes. These patients were categorized by frailty level into low-, intermediate-, and high-risk frailty. Differences in treatments and outcomes were explored in high-risk frailty patients and compared with those with intermediate- and low-risk frailty, with the primary exposure of interest being revascularization compared with medical management. The primary outcome was 1-year cardiovascular mortality. This research followed the STROBE guidelines for reporting observational studies.^14^

The study received appropriate governance approvals from the University of Leicester Research Ethics Committee and NHS Digital.

### Hospital Episode Statistics data set

The HES Admitted Patient Care (APC) data set containing information on all patients admitted to an NHS hospital in England was used. Each patient had an anonymized identifier, which allows longitudinal follow-up for all admissions from 2007 to 2020 and linkage to Office for National Statistics (ONS) mortality data. The data sets contained demographic and geographic data information, diagnoses (coded by ICD-10 codes), and procedures (coded by OPCS4 codes). Hospital Episode Statistics data have a primary diagnostic coding accuracy of 96% from 2002 onwards, while the coding of procedures is 97% accurate.^15,16^

### Cohort selection

Patients with an ICD-10 code (see Supplementary data online, *Table S1*) for ST elevation myocardial infarction (STEMI), non-STEMI (NSTEMI), or unstable angina were identified between 1 April 2010 and 31 March 2015 in the HES APC data set. The proportion of NSTEMI codes increased over the study time. On further review of the data set, the I21.9 (unspecified acute myocardial infarction) code decreased simultaneously, which was then included in the definition of the NSTEMI. For each patient, the ACS index date was defined as the episode start date of the first appearance of one of these ICD-10 codes during this period. Patients with a diagnosis code of unstable angina were re-categorized if they received a STEMI or NSTEMI code during another presentation within 6 months from the index date but retained their original index date for the analysis. For all included patients, information on all hospital admissions was collected for 2 years before their index date and at least 5-year follow-up.

### Frailty and comorbidities

Patient frailty risks were determined using the Hospital Frailty Risk Score (HFRS), previously developed and validated in a large HES data set. Consistent with the authors developing the score, patients were considered frail if they were in the intermediate-risk and high-risk frailty groups. The score included codes related to dementia, cognitive dysfunction, incontinence, senility, falls, or care dependency.^17^ Hospital Frailty Risk Score was calculated using ICD-10 codes for the comorbidities listed within 2 years before the index date presentation. As described in the original study,^17^ the following frailty categories were used: <5, low-risk frailty; 5–15, intermediate-risk frailty; and >15, high-risk frailty. Patients are only scored in the HFRS if they have an ICD-10 code. If they scored 0, they were placed in the low frailty category.

### Demographics

Information on sex, age, social deprivation (using the index of multiple deprivation), and patients’ residence (defined through the first letter of the postcode) was collected from the index episode. Due to missing data on ethnicity, all episodes in the 2 years before the index date with an entry were collated for each patient, and the most commonly used entry for each patient was selected.

### Interventions and treatments

A patient was deemed to have had an intervention or treatment if they had a relevant ACS OPCS4 code within 6 months of the index date to allow for delays caused by NHS waiting times. Investigations of interest included coronary angiography (K63, K64, and K65), echocardiogram (U20), and cardiac magnetic resonance imaging (U10.3 and U106.). All patients who underwent revascularization were considered to have had coronary angiography, given that some will have had revascularization at their primary angiogram without this being double coded for remuneration. Treatments of interest were percutaneous coronary intervention (PCI) (K49, K50, and K75), coronary artery bypass grafting (CABG) (K40–46), and medical management only (defined as the absence of PCI or CABG codes). Revascularization was defined as a composite of PCI and CABG. Patients were assigned to medical management if they did not receive a revascularization procedure within 6 months (considered long enough to undergo revascularization based on existing guidance^8,18^) of the index ACS date.

### Outcome variables

Cardiovascular mortality was the primary outcome. Date and cause of death were established from the linked ONS mortality data, with all-cause and cardiovascular mortality identified within 1-year and 5-year intervals from the index date. Stroke (I60–I66), acute myocardial infarction (I21), and readmission for ischaemic heart diseases (I20–I25) or heart failure (I11.0, I13.2, I13.0, and I50) were not defined by the order in which they occurred within a single episode; hence, in this analysis, these were defined as starting from the discharge date of the hospital spell. Bleeding and death were defined and determined from the index date. Major adverse cardiovascular event (MACE) was defined as a diagnosis of stroke, myocardial infarction, or cardiovascular mortality. These outcomes were calculated as a count and percentage stratified by frailty level.

Other outcomes related to hospital stay were death during the original hospital spell, length of first hospital spell, and days alive out of the hospital at 30 days. Days alive out of hospital was calculated with the ACS index date as Day 0. Each full day the patient spent discharged from the hospital during the following 30 days was counted. However, if the patient passed away during the in-hospital period, the number of days alive out of hospital was considered to be zero.

### Statistical analysis

#### Demographic information

Information on patient demographics, interventions, and outcomes were presented as numbers and percentages for categorical variables, with statistical significance tested using a *χ*^2^ test. Continuous variables were presented as mean and standard deviation if the data were normally distributed. Otherwise, as median and interquartile range, statistical significance was tested by *t*-test or Mann–Whitney *U* test, respectively.

#### Adjusted models for the likelihood of investigations and revascularization

Individual logistic models using the following binary variables as outcomes, angiography, echocardiogram, CABG, PCI, and medical management, were fitted to examine the effect of frailty level on the received interventions and treatments. The models were adjusted for, age, sex, ethnicity, social deprivation quintile, diagnosis (NSTEMI, STEMI, and unstable angina), and prior history of atrial fibrillation, diabetes, chronic obstructive pulmonary disease, stroke/transient ischaemic attack (TIA), coronary artery disease (CAD), congestive heart failure (CHF), chronic kidney disease (CKD), CABG/PCI, and history of pulmonary hypertension. A complete case analysis was used for all models. Due to 11 611 patients having missing data on some variables, 553 767 patients were included in the adjusted models. There was no significant difference in missing variables between frailty risk categories.

#### Outcomes

Standardized 5-year probabilities of freedom from cardiovascular death in all ACS patients with and without revascularization were estimated using Cox proportional hazards models stratified for different levels of frailty and adjusted for age, sex, diagnosis, social deprivation quintiles, and comorbidities. The modelling of cardiovascular-cause mortality was not adjusted for competing risks such as non-cardiovascular-cause death. Patients were censored at whichever came first: the date of death from any cause or the final data collection date for the study, 19 October 2020.

#### Geographical variation in revascularization

A logistic model was fitted using revascularization as the outcome variable. Revascularization was adjusted for age, sex, frailty, diagnosis, social deprivation quintiles, prior CAD, prior CHF, prior CKD, prior atrial fibrillation, prior diabetes, prior chronic obstructive pulmonary disease, prior stroke/TIA, prior CABG/PCI, and prior pulmonary hypertension. This model was then used to fit a prediction of the probabilities of each patient receiving revascularization based on the variables adjusted for in the model. The observed and predicted revascularization rates were then summed by outward postcode area. The revascularization ratio was then calculated by dividing each area’s observed and expected total. These were shown on a funnel plot (FunnelplotR, R-package) with 99.8% confidence intervals (CI), with any outward postcode areas outside these limits considered outliers.

#### Treatment effect estimates

Causal inference analysis using recursive bivariate probit (RBIProbit, STATA) instrumental variable models was used to estimate the effects of revascularization on cardiovascular mortality with adjustment for measured and unmeasured confounders.^19^ An instrumental variable should be associated with the treatment received but not with confounders and, therefore, only affects outcomes via the treatment received. Revascularization rate by postcode was chosen as the instrumental variable as it met these conditions. Models were stratified by frailty level and adjusted for known confounders, including age, sex, social deprivation quintiles, prior history of CAD, CHF, CKD, atrial fibrillation, diabetes, chronic obstructive pulmonary disease, stroke/TIA, CABG/PCI, and pulmonary hypertension. Effect estimates were expressed as average treatment effect (ATE) with 95% CI, which may be interpreted as absolute risk differences. Correlation between the revascularization rate and the instrumental variable was checked by comparing the first stage F-statistic with the Stock–Yogo critical value. This test is used to ensure that bias is less than 5%, 10%, or 30% of the worst-case error from ordinary least squares and determines the strength of the instrumental variable used. Demographic characteristics by quintile of the instrumental variable, stratified by frailty level, were assessed for evidence of a trend in values of measured confounders. A sensitivity analysis used known confounders and logistic regression with treatment effects expressed as odds ratios (OR) and ATEs with 95% CI to examine treatment effects unadjusted for unmeasurable confounding.

The statistical analyses were performed using R 4.2.3 and Stata 17 (Stata Corporation, Texas, USA).

## Results

**Figure 1:**
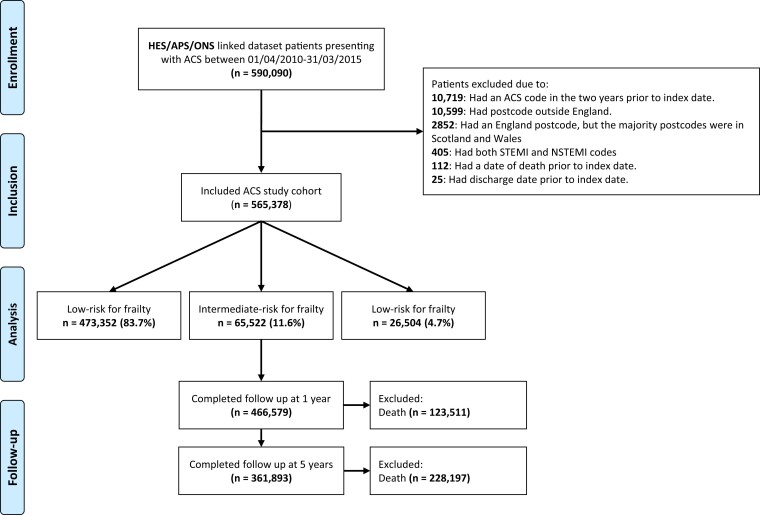
Strengthening the reporting of observational studies in epidemiology diagram detailing the included and excluded acute coronary syndrome patients stratified by risk for frailty and the number of patients completing follow-up at 1 and 5 years

Between April 2010 and March 2015, 590 090 patients with an ACS code were identified. From these, 24 712 patients were excluded from the cohort for the following reasons: 10 719 had an ACS code in the 2 years before the index date, 10 599 had a postcode outside England, 2852 had the majority of the postcodes in Scotland or Wales, 405 had both STEMI and NSTEMI codes, 112 had a date of death prior the index date, and 25 had a discharge date prior the index date (*Figure 1*). After exclusions, 565 378 patients were included in the analysis.

### Demographics

In the analysis cohort, 473 352 were low risk, 65 522 were intermediate risk, and 26 504 were high risk for frailty (83.7% vs. 11.6% vs. 4.7%). Low-risk frailty patients were younger (median age 70 vs. 81 vs. 84) and more likely to present with STEMI (28% vs. 16.7% vs. 13.7%) than intermediate- and high-risk frailty patients. High-risk frailty patients were more likely to be female than intermediate- and low-risk frailty (56.9% vs. 50.6% vs. 36.3%). High-risk frailty patients had higher levels of all comorbidities except prior CABG/PCI. Patients with high-risk frailty were more frequently diagnosed with NSTEMI than STEMI or unstable angina (59.2% vs. 13.7% vs. 27.1%) (*Table 1*). Differences in sex distribution, ethnicity, social deprivation, and comorbidities between the revascularized and non-revascularized groups or geographical variation in each frailty group are shown in Supplementary data online, *Tables S2*–*S5*.

**Table 1:** Differences in demographics and comorbidities presented by frailty level

	Low-risk frailty *N* = 473 352 (83.7%)	Intermediate-risk frailty *N* = 65 522 (11.6%)	High-risk frailty *N* = 26 504 (4.7%)
**Age, years**	70.0 (59.0–80.0)	81.0 (72.0–87.0)	84.0 (77.0–89.0)
**Sex**			
Male	301 559 (63.7)	32 367 (49.4)	11 424 (43.1)
Female	171 766 (36.3)	33 155 (50.6)	15 080 (56.9)
**Ethnicity**			
White	418 573 (90.5)	60 229 (92.6)	24 798 (94.1)
Black	6176 (1.3)	921 (1.4)	392 (1.5)
Asian	30 142 (6.5)	3233 (5.0)	909 (3.4)
Mixed/other	7527 (1.6)	628 (1.0)	261 (1.0)
**Social deprivation**			
Least deprived quintile	84 034 (17.8)	10 323 (15.8)	4013 (15.1)
2nd quintile	93 539 (19.8)	12 008 (18.3)	4775 (18.0)
3rd quintile	97 753 (20.7)	14 078 (21.5)	5745 (21.7)
4th quintile	97 154 (20.5)	14 253 (21.8)	5912 (22.3)
Most deprived quintile	100 872 (21.3)	14 860 (22.7)	6059 (22.9)
**Diagnosis**			
NSTEMI	220 359 (46.6)	36 237 (55.3)	15 685 (59.2)
STEMI	132 308 (28.0)	10 949 (16.7)	3643 (13.7)
Unstable angina	120 685 (25.5)	18 336 (28.0)	7187 (27.1)
**Admission status**			
Elective	17 914 (3.9)	2463 (3.9)	528 (2.0)
Emergency	440 224 (96.1)	61 248 (96.1)	25 335 (98.0)
**Comorbidities**			
Previous CAD	76 634 (16.2)	28 190 (43.0)	12 268 (46.3)
Previous CHF	8573 (1.8)	9392 (14.3)	6012 (22.7)
Previous CKD	9200 (1.9)	11 804 (18.0)	7357 (27.8)
Previous atrial fibrillation	26 695 (5.6)	17 947 (27.4)	9898 (37.3)
Previous diabetes	49 252 (10.4)	20 511 (31.3)	9059 (34.2)
Previous chronic obstructive pulmonary disease	24 862 (5.3)	13 973 (21.3)	6433 (23.4)
Previous TIA/stroke	6359 (1.3)	7616 (11.6)	6341 (23.9)
Previous CABG/PCI	8402 (1.8)	1408 (2.1)	330 (1.2)
Previous pulmonary hypertension	1294 (.3)	1277 (1.9)	733 (2.8)

Twenty-seven patients had missing information on sex, 11 589 had missing data on ethnicity, and 3458 had a code for both PCI and CABG in the same episode and were excluded from this table. A total of 17 666 patients either had missing information on admission status or were not classified as elective or emergency admission.

CABG, coronary artery bypass grafting; CAD, coronary artery disease; CHF, congestive heart failure; CKD, chronic kidney disease; NSTEMI, non-ST elevation myocardial infarction; PCI, percutaneous coronary intervention; STEMI, ST elevation myocardial infarction; TIA, transient ischaemic attack.

### Frailty risk, investigations, and treatment

**Figure 2:**
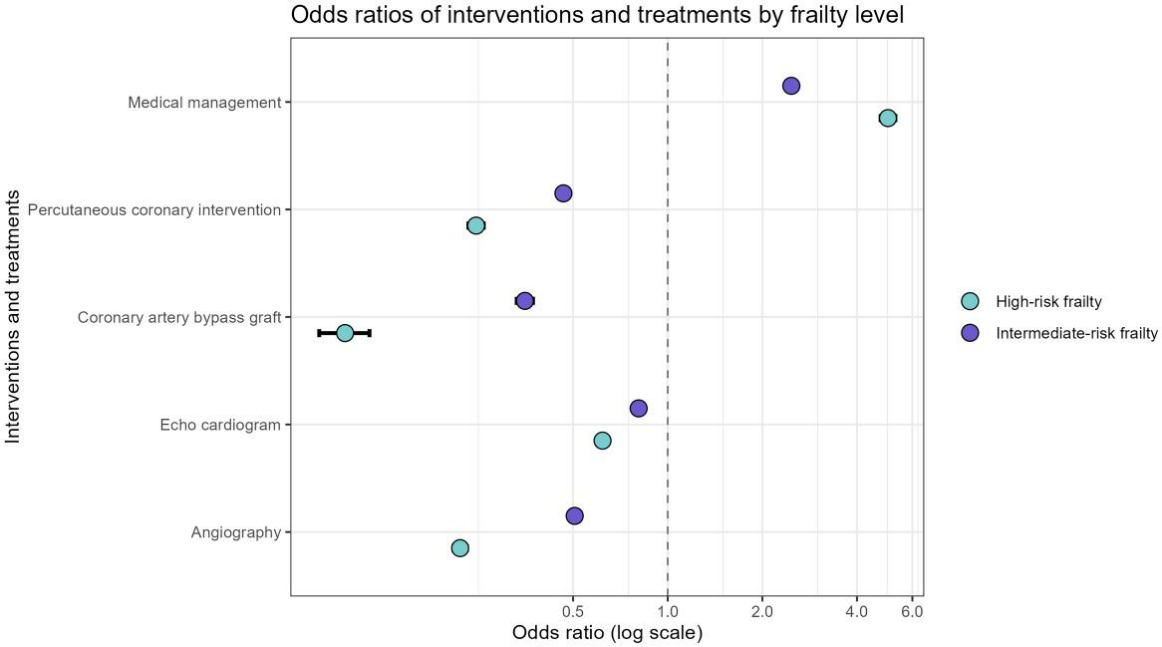
Likelihood of receiving intervention/treatment within 6 months from the index date, represented as odds ratios* and 95% confidence intervals. *Resultant odds ratios were adjusted for patients’ demographics, comorbidities, and acute coronary syndrome type. Low-risk frailty was used as the reference level in all models. CABG, coronary artery bypass grafting; PCI, percutaneous coronary intervention

Patients at high risk of frailty received fewer investigations and treatments than those at intermediate or low risk: angiography (11.9% vs. 27% vs. 65.8%, *P* < .001), echocardiography (29.6% vs. 35.8% vs. 44.4%, *P* < .001), CABG (.4% vs. 1.8% vs. 6.3%, *P* < .001), and PCI (5.9% vs. 13.4% vs. 40.3%, *P* < .001). Most high-risk frail patients (93.7%) were treated with medical management alone compared with 53% of low-risk frailty patients (*Table 2*). Logistic models adjusted for confounders confirmed that the differences in high-risk frailty patients compared with low-risk frailty patients were statistically significant (83% less likely to have angiography, 38% less likely to have echocardiography, 91% less likely to have CABG, and 75% less likely to have PCI) (*Figure 2* and *Table 2*; Supplementary data online, *Table S6*).

**Table 2:** Differences in investigations, treatments during index admission, and survival and clinical outcomes at index admission, 1 and 5 years stratified by frailty risk

	Low-risk frailty *N* = 473 352 (83.7%)	Intermediate-risk frailty *N* = 65 522 (11.6%)	High-risk frailty *N* = 26 504 (4.7%)	*P*-value
**Angiogram**	311 489 (65.8)	17 715 (27.0)	3141 (11.9)	<.001
**Echocardiogram**	210 057 (44.4)	23 458 (35.8)	7840 (29.6)	<.001
**Cardiac MRI**	6175 (1.3)	410 (.6)	59 (.2)	<.001
**CABG**	29 662 (6.3)	1200 (1.8)	113 (.4)	<.001
**PCI**	189 227 (40.3)	8745 (13.4)	1557 (5.9)	<.001
**Medical management**	251 105 (53.0)	55 482 (84.8)	24 829 (93.7)	<.001
**Total revascularizations**	222 247 (47.0)	10 040 (15.3)	1675 (6.3)	<.001
**Outcomes**				
Death during index ACS hospital stay	40 252 (8.5)	13 062 (19.9)	6655 (25.1)	<.001
Length of first hospital stay, days, median (IQR)	4 (2–8)	7 (3–16)	9 (4–20)	<.001
30 days alive out of hospital, median (IQR)	25 (19–27)	20 (9–26)	18 (5–25)	<.001
**One-year outcomes**				
One-year all-cause mortality	80 590 (17.0)	28 292 (43.2)	14 629 (55.2)	<.001
One-year cardiovascular mortality	68 761 (14.5)	23 238 (35.5)	11 779 (44.4)	<.001
Stroke	11 353 (2.4)	3380 (5.2)	2171 (8.2)	<.001
Myocardial infarction	28 106 (5.9)	4738 (7.2)	1711 (6.5)	<.001
MACE	98 997 (20.9)	28 394 (43.3)	14 064 (53.1)	<.001
Readmission for ACS	220 721 (46.6)	34 283 (52.3)	12 600 (47.5)	<.001
Readmission for heart failure	52 297 (11.0)	14 317 (21.9)	5997 (22.6)	<.001
Major bleeding	33 443 (7.1)	7628 (11.6)	3004 (11.3)	<.001
**Five-year outcomes**				
Five-year all-cause mortality	156 216 (33.0)	48 595 (74.2)	23 386 (88.2)	<.001
Five-year cardiovascular mortality	122 792 (25.9)	37 579 (57.4)	17 693 (66.8)	<.001
Stroke	28 842 (6.1)	7086 (10.8)	3564 (13.4)	<.001
Myocardial infarction	55 572 (11.7)	8676 (13.2)	2826 (10.7)	<.001
MACE	173 182 (36.6)	43 741 (66.8)	19 679 (74.2)	<.001
Readmission for ACS	326 942 (69.1)	42 488 (64.8)	14 789 (55.8)	<.001
Readmission for heart failure	101 153 (21.4)	22 641 (34.6)	8469 (32.0)	<.001
Major bleeding	66 237 (14.0)	12 823 (19.6)	4691 (17.7)	<.001

All values are *N* (%) unless otherwise stated. MACE was defined as a code for MI, stroke, or cardiovascular mortality. Interventions and treatments were identified within 6 months of initial admission for ACS; 3458 patients had an OPCS-4 code for a PCI and CABG within the same episode.

ACS, acute coronary syndrome; CABG, coronary artery bypass grafting; IQR, interquartile range; MACE, major adverse cardiovascular events; MRI, magnetic resonance imaging; PCI, percutaneous coronary intervention.

### Frailty risk and outcomes

Patients with intermediate and high frailty risk had higher mortality during their initial hospital stay, with 25.1%, 19.9%, and 8.5% of patients dying without being discharged from hospital for high-, intermediate-, and low-risk frailty patients, respectively. Length of initial hospital stay was longer for the frailer patients, with a median stay of 9 days for high-risk frailty patients, 7 for intermediate, and 4 for low-risk frailty. Days alive and out of the hospital (30 days) had a median of 18 days for high-risk frailty, 20 days for intermediate-risk frailty, and 25 days for low-risk frailty (*Table 2*).

**Figure 3:**
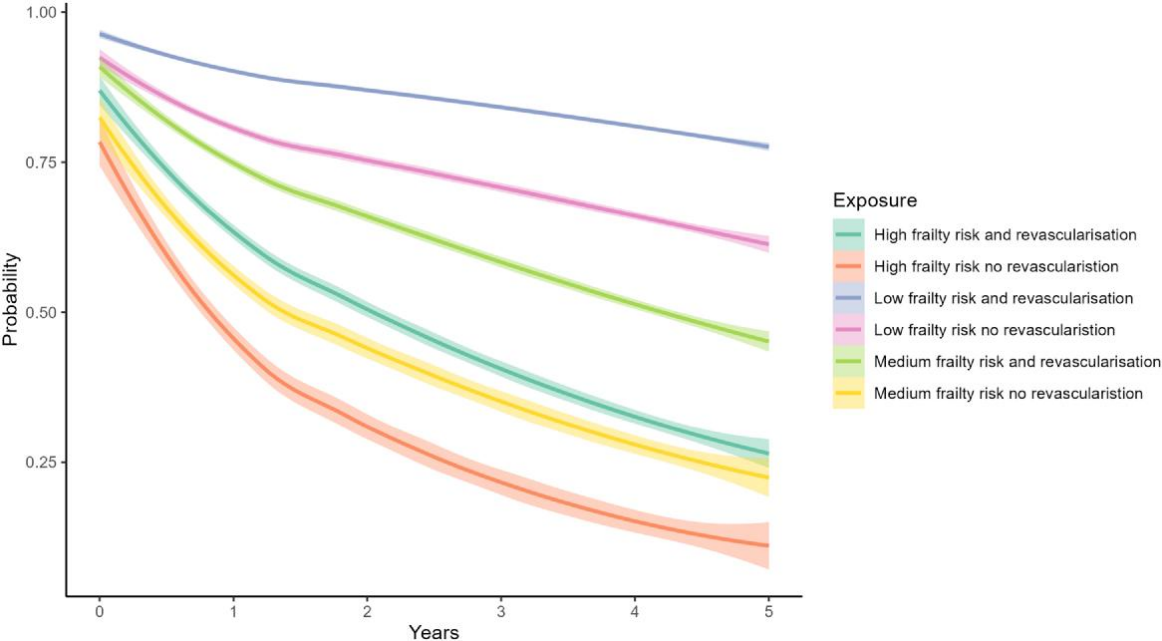
Kaplan–Meier curves showing the 5-year predicted survival stratified by frailty level and revascularization status

People at intermediate and high risk of frailty demonstrated incremental increases in mortality in hospital at 1 year and 5 years post ACS, compared with those at low risk (*Figure 3* and *Table 2*). One-year all-cause mortality was 17%, 43.2%, and 55.2% for low-, intermediate-, and high-risk groups, respectively. Five-year all-cause mortality was 33%, 74.2%, and 88.2% for low-, intermediate-, and high-risk groups, respectively. Cardiovascular causes were the principal mode of death across all strata and accounted for 78.6%, 77.3%, and 75.7% of deaths at 5 years in people at low, intermediate, and high frailty risk, respectively. Competing non-cardiovascular causes of death are shown in Supplementary data online, *Table S7*, and competing causes contributing to cardiovascular mortality are listed in Supplementary data online, *Table S8*. Over 5 years of follow-up, people at intermediate or high risk of frailty experienced incrementally higher rates of stroke, MACE and readmissions with heart failure or major bleeding, and lower rates of readmission with ACS compared with those at low risk (*Table 2*).

**Figure 4:**
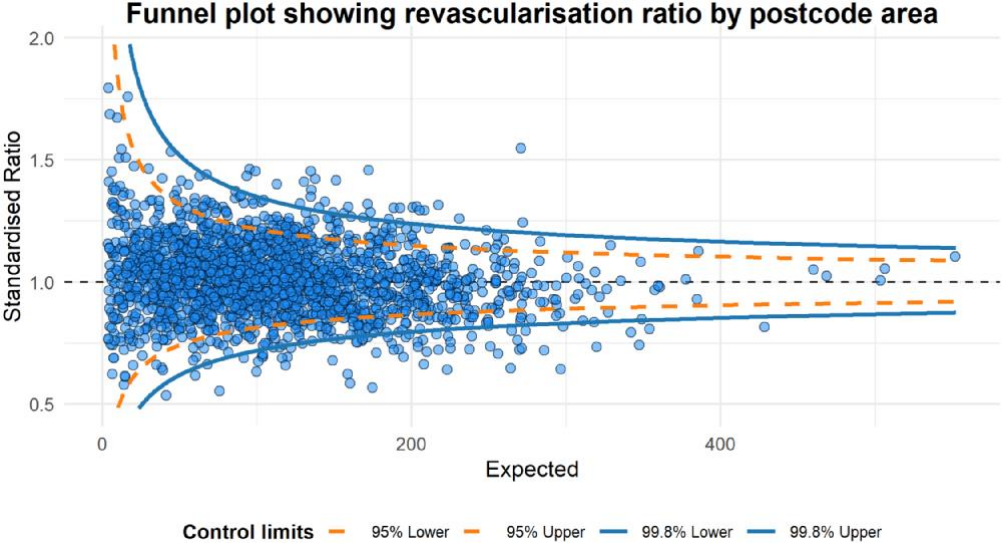
Funnel plot demonstrating the revascularization rate by outward postcode. Using a 99.8% confidence limit, 97 of the 2024 areas were outliers, which demonstrated the geographical variation of revascularization rates

The revascularization rate for all patients, irrespective of degree of frailty, varied from 16.3% to 78.9% by postcode area, with a median of 41.7% (IQR: 37.1%–46.7%) (*Figure 4*).

### Geographical variation in revascularization

**Figure 5:**
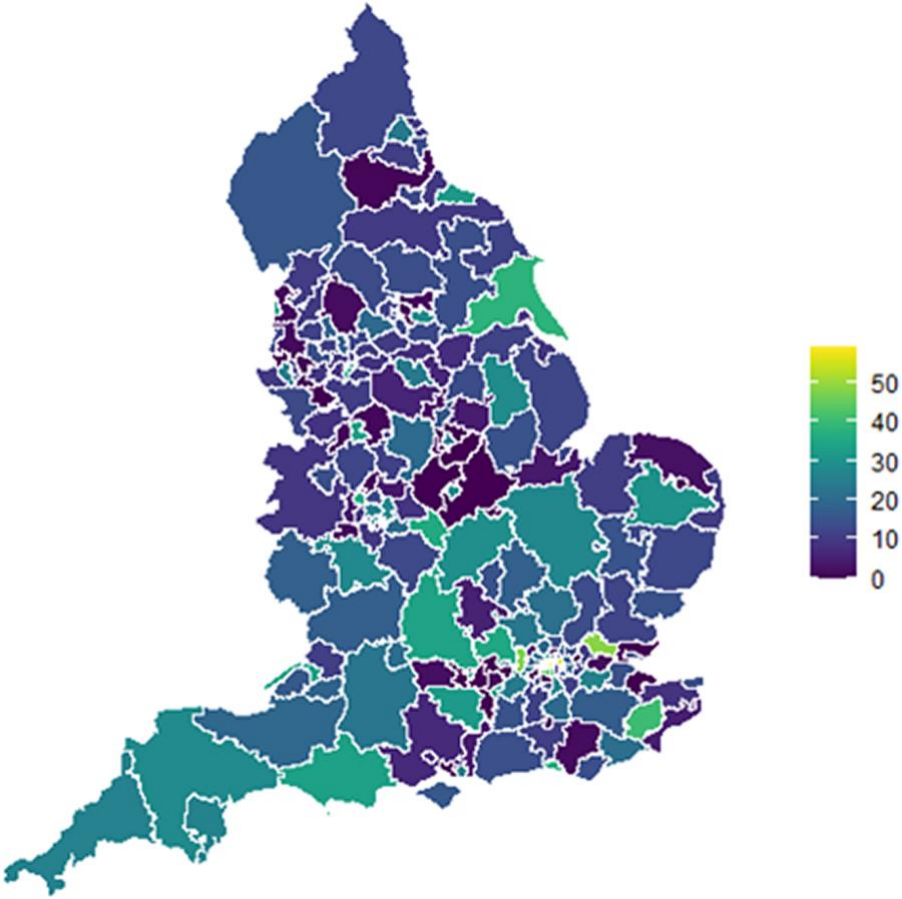
Heatmap of revascularization percentage rates by postcode showing variation in revascularization; .05% of the areas were outside the 99.8% confidence limits and were considered outliers. Colour coding based on the percentage of revascularizations, with purple being closest to 0% and yellow to 50%

Funnel plots were fitted with outward postcodes as the grouping factor. Ninety-seven out of 2024 (.05%) areas were outside the 99.8% confidence limits and were considered outliers, indicating unwarranted variation in revascularization rates that was not explained by patient-level factors (*Figure 5*). The regional revascularization rate was independent of known confounders and was associated with and correlated with the primary outcome (*Table 3*; Supplementary data online, *Table S9*).

**Table 3:** Logistic regression and instrumental variable analyses showing the effect of revascularisation vs. no revascularization stratified by frailty level on 1-year and 5-year cardiovascular mortality

	Patients revascularized *N* (%)	Logistic regression		Bivariate probit IV, ATE (95% CI)
		OR (95% CI)	ATE (95% CI)	
**One-year cardiovascular mortality**				
Low	222 022 (46.9)	.24 (.23–.24)	−.13 (−.13 to −.13)	−.13 (−.14 to −.13)
Intermediate	10 034 (15.3)	.30 (.29–.32)	−.22 (−.23 to −.21)	−.22 (−.23 to −.21)
High	1675 (6.3)	.47 (.42–.53)	−.18 (−.21 to −.15)	−.20 (−.23 to −.17)
**Five-year cardiovascular mortality**				
Low	222 022 (46.9)	.32 (.32–.33)	−.16 (−.16 to −.16)	−.16 (−.16 to −.16)
Intermediate	10 034 (15.3)	.44 (.42–.46)	−.17 (−.18 to −.16)	−.18 (−.19 to −.17)
High	1675 (6.3)	.68 (.61–.76)	−.07 (−.10 to −.04)	−.10 (−.13 to −.06)

Due to missing data on some variables, 553 767 patients were included in the fitted models. All models were adjusted for age, sex, quintile of deprivation, ethnicity, diagnoses, and prior comorbidities. Cardiovascular mortality at 1 and 5 years (presented as absolute value and percentage): low risk of frailty, 68 761 (14.5) and 122 792 (25.9); intermediate risk of frailty, 23 238 (35.5) and 37 579 (57.4); high risk of frailty, 11 779 (44.4) and 17 693 (66.8).

ATE, average treatment effect; CI, confidence interval; OR, odds ratio.

### Effect of revascularization on 1- and 5-year cardiovascular mortality

In evaluating the revascularization rate as the statistical instrument, the first stage F-statistic was 80.8 (*P* < .001) and was greater than the Stock–Yogo critical value of 16.4 at the 10% level for high-risk frailty patients. For low and intermediate frailty risk, the first stage F-statistic was 5664.2 (*P* < .001) and 605.1 (*P* < .001), indicating that the revascularization rate by outward postcode area was a strong instrumental variable.

Next, the baseline characteristics of the cohort were examined separately for each frailty level stratified by quintiles of the instrumental variable. There was evidence of an increasing proportion of the patients being male across quintiles. However, the other demographic variables, including the social deprivation index, were well balanced (see Supplementary data online, *Table S5*).

**Figure 6:**
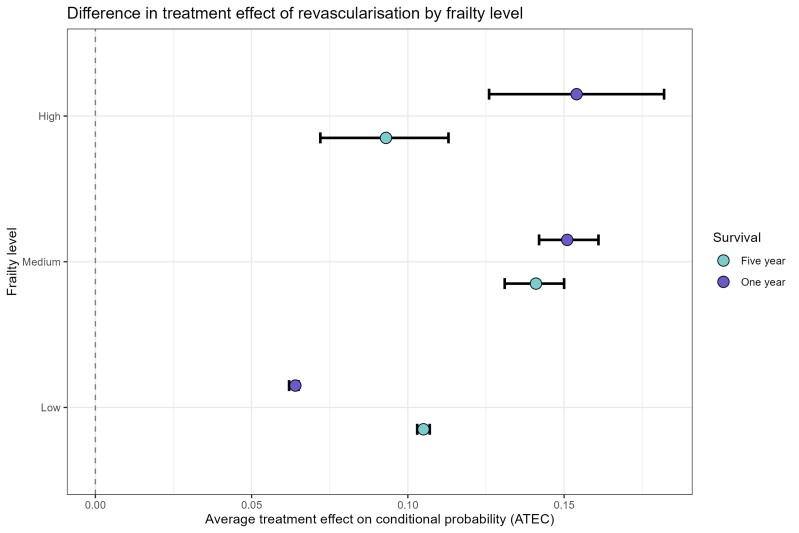
Instrumental variable analysis showing the difference in the effects of revascularization on 1-year and 5-year cardiovascular mortality based on frailty levels. The results are presented as average treatment effects and 95% confidence intervals

Using the regional revascularization rate as the instrumental variable, the bivariate probit model showed that revascularization was associated with the smallest absolute reduction in 1-year cardiovascular death in low-frailty risk, ATE −13% (95% CI −14% to −13%), vs. the intermediate, −22% (95% CI −23% to −21%), and high frailty risk groups, −20% (95% CI −23% to −17%) (*Table 3*; Supplementary data online, *Table S9*). At 5 years, this was reversed, although the reduction in mortality was still statistically different for all categories with ATE of −16% (95% CI −16% to −16%), −18% (95% CI −19% to −17%), and −10% (95% CI −13% to −6%) for low, intermediate, and high frailty risk, respectively (*Table 3*; Supplementary data online, *Table S10*). In sensitivity analyses, logistic regression analyses, which were not adjusted for unmeasured confounding, demonstrated similar results when compared with ATE (*Figure 6*).

## Discussion

### Main findings

Our longitudinal observational analysis of 565 378 people presenting with ACS demonstrated that over 1 in 6 were at intermediate or high risk for frailty. These patients experienced significantly fewer investigations and interventions and had worse outcomes than those at low risk for frailty. Cardiovascular causes were the leading cause of death in ACS patients with or without frailty. Causal inference analysis using regional variation in treatment as an instrumental variable demonstrated that revascularization was associated with significant survival benefits at 1 and 5 years in people with intermediate- or high-risk frailty (*Structured Graphical Abstract*).

### Research into context

There is a knowledge gap in trials evaluating people with frailty presenting with ACS.^20^ Guidelines recommend revascularization decisions considering baseline status and procedural risk, while tools for assessing frailty and indications are not specified.

The best evidence available is from RCTs of revascularization in older people presenting with ACS. However, these have shown conflicting results. The Elderly ACS trial^21^ in people with ACS over 70 years old showed no difference in mortality, myocardial infarction, and repeat hospital stay between an initial invasive vs. an initial conservative strategy at 1 year. In contrast, the After Eighty trial^22^ showed lower MACE at 1.5 years in people over 80 years old presenting with NSTEMI or unstable angina treated with an invasive vs. a conservative strategy. The proportions of frail patients in these trials were not specified, and they were highly selective, recruiting 48.5% and 10.9% of eligible patients, respectively. This was associated with lower rates of multimorbidity than those observed in the current analysis. The LONGEVO-SCA study^23^ demonstrated that frail patients were more likely to undergo conservative than invasive treatment. However, an invasive strategy led to no significant difference in clinical outcomes at 6 months in frail patients, which may be related to the use of different frailty, assessment, a limited sample size, a small number of events, and the 6-month follow-up, which is not sufficient to detect the impact of treatment strategy on clinical outcomes fully. When comparing invasive and conservative strategies, the MOSCA-FRAIL^24^ showed no significant increase in DAOH at 1 year. This study was terminated early due to the COVID-19 pandemic, had a higher crossover rate, and had a higher mean age (86 years), potentially leading to more complications with an invasive strategy. The Senior PAMI trial in people over 70 years who presented with STEMI also showed that revascularization with PCI resulted in a lower rate of death, disabling stroke, or reinfarction when comparing PCI with intravenous thrombolytic therapy. However, the follow-up was only 30 days, this effect was absent in >80-year-olds, and the trial was terminated early due to slow recruitment.^25^ Together, these results demonstrate the challenges of undertaking RCTs in this vulnerable group. Other factors usually not included in trials and routinely collected data are the type of vascular access, previous revascularization undertaken, patient decision-making, insurance, and health provision facilities, which may impact the outcomes of revascularization in high-risk frailty patients with ACS.

In the absence of RCTs, observational analysis at low risk of bias can inform treatment decisions. We have shown three novel findings using a novel study design that adjusts for important bias in observational analyses. First, as the population ages, the number of people presenting with frailty and ACS will likely increase. This study suggests that revascularization significantly reduces key clinical outcomes in this vulnerable group. Second, cardiovascular causes were the principal mode of death (>75%) following ACS, regardless of frailty risk. This refutes the suggestion that frail people with ACS die of non-cardiac causes and provides important new information for shared decision-making. This provides a rationale for further evaluation of cardiovascular interventions in this increasing population. Third, the knowledge gap and clinical uncertainty shown by regional variation in treatment across frailty risk groups demonstrate the unmet need for further high-quality research that considers the effects of treatment decisions on multiple frailty domains and processes, behaviours, and values underpinning complex treatment decisions.

### Strengths and limitations

The study has several strengths. First, using routinely collected health data from all English hospitals will likely mitigate potential selection bias. A major strength of HES data is that it captures 100% of hospital admissions as required for healthcare cost reimbursements and is >95% accurate for procedure codes and outcomes.^26^ The large sample size also reduces the likelihood that selection bias will have influenced our results, given that significant numbers of all important clinical subgroups and exposures will have been included in the analyses.

Second, the longitudinal follow-up provides important new information on mid- to long-term outcomes in this high-risk group, traditionally under-represented in cardiovascular research.

Third, we used causal inference analyses to overcome important biases in observational studies,^27^ including bias by indication, which is particularly important in this context. Decision-making by clinicians caring for patients with ACS is complex and based on multiple measured and unmeasured factors (confounders), including whether the treatment was withheld by medical staff or declined by patients, the sickest patients being refused treatment, coronary anatomy, left ventricular function, prognosis, and the perception of fitness. We attempted to mitigate these biases using instrumental variable analysis, a two-stage regression used to adjust for known and unknown confounders in observational analyses. The first stage of regression is between the instrument variable and treatment selection. The second stage of regression uses the model built in the first stage to predict outcomes based on treatment selection. A good instrument variable should strongly correlate with the treatment allocation and not be associated with measured and unmeasured patient characteristics. The effect of an instrumental variable on the outcome measure is then entirely mediated via its effect on treatment assignment. In this case, the variation of participants undergoing revascularization across regions in England served as a suitable instrument, rather than exposure to treatment yes/no as used in logistic regression. By demonstrating no apparent association between the instrumental variable and measured confounders, it can be inferred that associations with unmeasured confounders are unlikely. Our results were confirmed by logistic regression sensitivity analyses that did not adjust for unmeasured confounders. However, residual confounding factors related to HES health databases cannot be excluded.

The study has important limitations. First, the assessment of frailty using the HFRS, a validated database-derived frailty measure based on ICD-10 diagnostic codes in routinely collected electronic health records,^17^ will not have captured many important elements of frailty, including functional status, physical performance, cognition, or social isolation. Hospital Frailty Risk Score precision is also influenced by operator input, data accuracy and completeness, and variations in coding practices over time and across geographical regions.^28^ In contrast, clinical frailty assessments (e.g. Clinical Frailty Scale and Fried frailty phenotype) that capture multiple frailty domains are more time and resource demanding and unsuitable for large epidemiological analyses. Here, the HFRS and its limitations, notwithstanding, have enabled objective frailty screening in a large population-level cohort and provided novel insights into a target population that is not well represented in revascularization or clinical guidelines trials.

Second, the analysis did not include important outcomes like patient values, quality of life, ability to live independently, and cognition or physical activity. These factors are particularly important when assessing outcomes in frailty, whereas the current analysis only presents data on the effect of revascularization on major clinical events. Quality of life assessments and measures of frailty burden should be included in future trials in frail patients with ACS. Information on the complexity of coronary disease assessed by SYNTAX score or disease severity, use of dual antiplatelet therapy, and the number of treated vessels/lesions were also unavailable in the HES data set, which are additional important considerations for decision-making.

Third, revascularization rates in this sample were low compared with other reported series.^29,30^ Coronary revascularization rates in the UK are lower than the average in countries from the Organisation for Economic Co-operation and Development.^31^ The generalizability of these findings to countries with much higher revascularization rates may be limited.

## Conclusions

Frailty is common in people presenting with ACS, where cardiovascular causes are the principal mode of death. Revascularization is associated with short- and long-term survival benefits in people at intermediate and high risk of frailty after adjustment for measured and unmeasured confounders. Further research is required to validate these findings within the broader context of complex healthcare needs and priorities for this vulnerable and increasing patient group.

## Supplementary data

Supplementary data are available at *European Heart Journal* online.

## Supplementary Material

ehae755_Supplementary_Data

## Declarations

### Data Availability

NHS Digital provided the data underlying this article under licence. The data supporting this study’s findings are available from the corresponding author upon reasonable request. Raw data may be shared with the permission of NHS Digital.

### Pre-registered Clinical Trial Number

None supplied.
