# Supplementary material for: Revascularization in frail patients with acute coronary syndromes: a retrospective longitudinal study
Source: Eur Heart J. 2024 Nov 16;46(6):535–47. doi: 10.1093/eurheartj/ehae755 (PMC11804245; doi:10.1093/eurheartj/ehae755)
Supplement: ehae755_Supplementary_Data [file ehae755_supplementary_data.docx]

Supplementary Appendix

**Table S1.** ICD-10 Code lists

**Cohort definition**

|  | **ICD-10 code** | **Description** |
| --- | --- | --- |
| **STEMI** | I21.0 | Acute transmural myocardial infarction of interior wall |
|  | I21.1 | Acute transmural myocardial infarction of inferior wall |
|  | I21.2 | Acute transmural myocardial infarction of other sites |
|  | I21.3 | Acute transmural myocardial infarction of unspecified site |
|  | I22.0 | Subsequent myocardial infarction of anterior wall |
|  | I22.1 | Subsequent myocardial infarction of inferior wall |
|  | I22.8 | Subsequent myocardial infarction of other sites |
|  | I22.9 | Subsequent myocardial infarction of unspecified site |
| **NSTEMI** | I21.4 | Acute subendocardial myocardial infarction |
|  | I21.9 | Acute myocardial infarction, unspecified |
| **Unstable angina** | I20.0 | Unstable angina |

**Prior Comorbidities**

| **Comorbidity** | **ICD-10 or OPCS4 code** | **Description** |
| --- | --- | --- |
| **CAD** | ICD-10 I21 | Acute myocardial infarction |
|  | ICD-10 I22 | Subsequent myocardial infarction |
|  | ICD-10 I23 | Certain current complications following acute myocardial infarction |
|  | ICD-10 I24 | Other acute ischaemic heart diseases |
|  | ICD-10 I25 | Chronic ischaemic heart disease |
| **CHF** | ICD-10 I50.0 | Congestive heart failure |
| **CKD** | ICD-10 N18 | Chronic kidney disease |
| **Atrial fibrillation** | ICD-10 I48 | Atrial fibrillation and flutter |
| **Diabetes** | ICD-10 E10 | Type 1 diabetes mellitus |
|  | ICD-10 E11 | Type 2 diabetes mellitus |
|  | ICD-10 E12 | Malnutrition-related diabetes mellitus |
|  | ICD-10 E13 | Other specified diabetes mellitus |
|  | ICD-10 E14 | Unspecified diabetes mellitus |
| **COPD** | ICD-10 J40 | Bronchitis, not specified as acute or chronic |
|  | ICD-10 J41 | Simple and mucopurulent chronic bronchitis |
|  | ICD-10 J42 | Unspecified chronic bronchitis |
|  | ICD-10 J43 | Emphysema |
|  | ICD-10 J44 | Other chronic obstructive pulmonary disease |
| **TIA/stroke** | ICD-10 G45 | Transient cerebral ischaemic attacks and related syndromes |
|  | ICD-10 I60 | Subarachnoid haemorrhage |
|  | ICD-10 I61 | Intracerebral haemorrhage |
|  | ICD-10 I62 | Other nontraumatic intracranial haemorrhage |
|  | ICD-10 I63 | Cerebral infarction |
|  | ICD-10 I64 | Stroke, not specified as haemorrhage or infarction |
|  | ICD-10 I65 | Occlusion and stenosis of precerebral arteries, not resulting in cerebral infarction |
|  | ICD-10 I66 | Occlusion and stenosis of cerebral arteries, not resulting in cerebral infarction |
|  | ICD-10 I69 | Sequelae of cerebrovascular disease |
| **CABG/ PCI** | OPCS4 K40 | Saphenous vein graft replacement of coronary aftery |
|  | OPCS4 K41 | Other autograft replacement of coronary artery |
|  | OPCS4 K42 | Allograft replacement of coronary artery |
|  | OPCS4 K43 | Prosthetic replacement of coronary artery |
|  | OPCS4 K44 | Other replacement of coronary artery |
|  | OPCS4 K45 | Connection of thoracic artery to coronary artery |
|  | OPCS4 K46 | Other bypass of coronary artery |
|  | OPCS4 K49 | Transluminal balloon angioplasty of coronary artery |
|  | OPCS4 K50 | Other therapeutic transluminal operations on coronary artery |
|  | OPCS4 K75 | Percutaneous transluminal balloon angioplasty and insertion of stent into coronary artery |
| **MI** | ICD-10 I21 | Acute myocardial infarction |
|  | ICD-10 I22 | Subsequent myocardial infarction |
|  | ICD-10 I23 | Certain current complications following acute myocardial infarction |
| **Pulmonary hypertension** | ICD-10 127.0 | Primary pulmonary hypertension |
|  | ICD-10 127.1 | Kyphoscoliotic heart disease |
|  | ICD-10 127.2 | Other secondary pulmonary hypertension |

**Treatments**

|  | **OPCS4 Code** | **Description** |
| --- | --- | --- |
| **Angiography** | K63 | Contrast radiology of heart |
|  | K64 | Percutaneous operations on heart |
|  | K65 | Catheterisation of heart |
| **Echocardiogram** | U20 | Diagnostic echocardiography |
| **Cardiac MRI** | U10.3 | Cardiac magnetic resonance imaging |
|  | U10.6 | Myocardial perfusion scan |
| **PCI** | K49 | Transluminal balloon angioplasty of coronary artery |
|  | K50 | Other therapeutic transluminal operations on coronary artery |
|  | K75 | Percutaneous transluminal balloon angioplasty and insertion of stent into coronary artery |
| **CABG** | K40 | Saphenous vein graft replacement of coronary artery |
|  | K41 | Other autograft replacement of coronary artery |
|  | K42 | Allograft replacement of coronary artery |
|  | K43 | Prosthetic replacement of coronary artery |
|  | K44 | Other replacement of coronary artery |
|  | K45 | Connection of thoracic artery to coronary artery |
|  | K46 | Other bypass of coronary artery |

**Outcomes**

|  | **ICD-10 codes** | **Desciption** |
| --- | --- | --- |
| **Cardiovascular mortality**  **Stroke** | I | Diseases of the circulatory system |
|  | I60 | Subarachnoid haemorrhage |
|  | I61 | Intracerebral haemorrhage |
|  | I62 | Other nontraumatic intracranial haemorrhage |
|  | I63 | Cerebral infarction |
|  | I64 | Stroke, not specified as haemorrhage or infarction |
|  | I65 | Occlusion and stenosis of precerebral arteries, not resulting in cerebral infarction |
|  | I66 | Occlusion and stenosis of cerebral arteries, not resulting in cerebral infarction |
| **MI** | I21 | Acute myocardial infarction |
| **Readmission for ACS** | I20 | Angina pectoris |
|  | I21 | Acute myocardial infarction |
|  | I22 | Subsequent myocardial infarction |
|  | I23 | Certain current complications following acute myocardial infarction |
|  | I24 | Other acute ischaemic heart diseases |
|  | I25 | Chronic ischaemic heart disease |
| **Readmission for Heart Failure** | I11 |  |
|  | I13.0 | Hypertensive heart and renal disease with (congestive) heart failure |
|  | I13.2 | Hypertensive Heart and Chronic Kidney Disease With Heart Failure and With Stage 5 Chronic Kidney Disease, or End Stage Renal Disease. |
|  | I50 | Heart Failure |
| **Major bleeding** |  |  |
| **GI Bleeding** | K92.0 | Haematemesis |
|  | K92.1 | Melaena |
|  | K92.2 | Gastrointestinal haemorrhage, unspecified |
|  | K22.8 | Other specified diseases of oesophagus |
| **Ulcer related bleeding** | K25.0 | Gastric ulcer-Acute with haemorrhage |
|  | K25.2 | Gastric ulcer-Acute with haemorrhage and perforation |
|  | K25.4 | Gastric ulcer-Chronic or unspecified with haemorrhage |
|  | K25.6 | Gastric ulcer- Chronic or unspecified with both haemorrhage and perforation |
|  | K26.0 | Duodenal ulcer-Acute with haemorrhage |
|  | K26.2 | Duodenal ulcer-Acute with haemorrhage and perforation |
|  | K26.4 | Duodenal ulcer-Chronic or unspecified with haemorrhage |
|  | K26.6 | Duodenal ulcer-Chronic or unspecified with both haemorrhage and perforation |
|  | K27.0 | Peptic ulcer, site unspecified-Acute with haemorrhage |
|  | K27.2 | Peptic ulcer, site unspecified-Acute with both haemorrhage and perforation |
|  | K27.4 | Peptic ulcer, site unspecified-Chronic or unspecified with haemorrhage |
|  | K27.6 | Peptic ulcer, site unspecified- Chronic or unspecified with both haemorrhage and perforation |
|  | K28.0 | Gastrojejunal ulcer-Acute with haemorrhage |
|  | K28.2 | Gastrojejunal ulcer-Acute with haemorrhage and perforation |
|  | K28.4 | Gastrojejunal ulcer-Chronic or unspecified with haemorrhage |
|  | K28.6 | Gastrojejunal ulcer-Chronic or unspecified with both haemorrhage and perforation |
| **Gastritis related bleeding** | K29.0 | Acute haemorrhagic gastritis |
|  | K62.5 | Haemorrhage of anus and rectum |
|  | K64 | Haemorrhoids and perianal venous thrombosis |
|  | K66.1 | Haemoperitoneum |
|  | K85 | Acute pancreatitis |
|  | K92.2 | Gastrointestinal haemorrhage, unspecified |
|  | I85.0 | Oesophageal varices with bleeding |
|  | I98.3 | Oesophageal varices with bleeding in diseases classified elsewhere |
| **Airway bleeding** | R04 | Haemorrhage from respiratory passages |
| **Cerebral bleeding** | I60 | Subarachnoid haemorrhage |
|  | I61 | Intracerebral haemorrhage |
|  | I62 | Other nontraumatic intracranial haemorrhage |
| **Urological bleeding** | R31 | Unspecified haematuria |
|  | N02 | Recurrent and persistent haematuria |
| **Gynae bleeding** | N93.9 | Abnormal uterine and vaginal bleeding, unspecified |
|  | O72 | Postpartum haemorrhage |
|  | O67 | Labour and delivery complicated by intrapartum haemorrhage, not elsewhere classified |
| **Pericardial effusion** | I312 | Haemopericardium, not elsewhere classified |
|  | I313 | Pericardial effusion (noninflammatory) |
| **Pleural effusion (Haemothorax)** | J94.2 | Haemothorax |
| **Haemorrhagic conditions/disorders** | D683 | Haemorrhagic disorder due to circulating anticoagulants |
|  | D698 | Other specified haemorrhagic conditions |
| **Other bleeding** | I230 | Haemopericardium as current complication following acute myocardial infarction |
|  | I312 | Haemopericardium, not elsewhere classified |
|  | S064 | Epidural haemorrhage |
|  | M250 | Haemarthrosis |
|  | T828 | Other specified complications of cardiac and vascular prosthetic devices, implants and grafts |
|  | R58 | Haemorrhage, not elsewhere classified |
| **Inherited bleeding disorders to be excluded** | D65 | Disseminated intravascular coagulation [defibrination syndrome] |
|  | D66 | Hereditary factor VIII deficiency |
|  | D67 | Hereditary factor IX deficiency |
|  | D68 | Other coagulation defects |
|  | D69 | Purpura and other haemorrhagic conditions |

**Table S2.** Demographics and comorbidities by frailty level and whether patients underwent or not revascularisation in each group.

|  | | | Low frailty risk | | | Intermediate frailty risk | | | High frailty risk | | |
| --- | --- | --- | --- | --- | --- | --- | --- | --- | --- | --- | --- |
|  | No revascularisation  N = 251,105 | Revascularisation  N = 222,247 | | P-value | No revascularisation N = 55,482 | Revascularisation N = 10,040 | P-value | No revascularisation N = 24,829 | | Revascularisation N = 1,675 | P-value |
| Age | 76.0 (64.0-84.0) | 65.0 (55.0-74.0) | | <0.001 | 82.0 (74.0-88.0) | 74.0 (65.0-81.0) | <0.001 | 84.0 (77.0-89.0) | | 76.0 (68.0-83.0) | <0.001 |
| Sex |  |  | | <0.001 |  |  | <0.001 |  | |  | <0.001 |
| Male | 136,125 (54.2) | 165,434 (74.4) | |  | 26,180 (47.2) | 6,187 (61.6) |  | 10,494 (42.3) | | 930 (55.5) |  |
| Female | 114,962 (45.8) | 56,804 (25.6) | |  | 29,302 (52.8) | 3,853 (38.4) |  | 14,335 (57.7) | | 745 (44.5) |  |
| Ethnicity |  |  | | <0.001 |  |  | <0.001 |  | |  | <0.001 |
| White | 224,051 (91.3) | 194,522 (89.6) | |  | 51,344 (93.4) | 8,885 (88.7) |  | 23,299 (94.4) | | 1,499 (89.7) |  |
| Black | 3,887 (1.6) | 2,289 (1.1) | |  | 778 (1.4) | 143 (1.4) |  | 361 (1.5) | | 31 (1.9) |  |
| Asian | 13,812 (5.6) | 16,330 (7.5) | |  | 2,386 (4.3) | 847 (8.5) |  | 785 (3.2) | | 124 (7.4) |  |
| Mixed/other | 3,665 (1.5) | 3,862 (1.8) | |  | 487 (0.9) | 141 (1.4) |  | 243 (1.0) | | 18 (1.1) |  |
| Social deprivation |  |  | | <0.001 |  |  | 0.015 |  | |  | 0.047 |
| Least deprived quintile | 43,460 (17.3) | 40,574 (18.3) | |  | 8,707 (15.7) | 1,616 (16.1) |  | 3,778 (15.2) | | 235 (14.0) |  |
| 2^nd^ quintile | 49,383 (19.7) | 44,156 (19.9) | |  | 10,279 (18.5) | 1,729 (17.2) |  | 4,505 (18.1) | | 270 (16.1) |  |
| 3^rd^ quintile | 52,391 (20.9) | 45,362 (20.4) | |  | 11,849 (21.4) | 2,229 (22.2) |  | 5,388 (21.7) | | 357 (21.3) |  |
| 4^th^ quintile | 51,954 (20.7) | 45,200 (20.3) | |  | 12,094 (21.8) | 2,159 (21.5) |  | 5,510 (22.2) | | 402 (24.0) |  |
| Most deprived quintile | 53,917 (21.5) | 46,955 (21.1) | |  | 12,553 (22.6) | 2,307 (23.0) |  | 5,648 (22.7) | | 411 (24.5) |  |
| Diagnosis |  |  | | <0.001 |  |  | <0.001 |  | |  | <0.001 |
| Non-ST elevation myocardial infarction (NSTEMI) | 123,645 (49.2) | 96,714 (43.5) | |  | 31,399 (56.6) | 4,838 (48.2) |  | 14,877 (59.9) | | 808 (48.2) |  |
| ST elevation myocardial infarction (STEMI) | 35,331 (14.1) | 96,977 (43.6) | |  | 7,172 (12.9) | 3,777 (37.6) |  | 2,944 (11.9) | | 688 (41.1) |  |
| Unstable angina | 92,129 (36.7) | 28,556 (12.8) | |  | 16,911 (30.5) | 1,425 (14.2) |  | 7,008 (28.2) | | 179 (10.7) |  |
| Comorbidities |  |  | |  |  |  |  |  | |  |  |
| Previous coronary artery disease (CAD) | 50,182 (20.0) | 26,452 (11.9) | | <0.001 | 23,976 (43.2) | 4,214 (42.0) | 0.021 | 11,463 (46.2) | | 805 (48.1) | 0.140 |
| Previous congestive heart failure (CHF) | 7,286 (2.9) | 1,287 (0.6) | | <0.001 | 8,608 (15.5) | 784 (7.8) | <0.001 | 5,745 (23.1) | | 267 (15.9) | <0.001 |
| Previous chronic kidney disease (CKD) | 6,718 (2.7) | 2,482 (1.1) | | <0.001 | 9,977 (18.0) | 1,827 (18.2) | 0.616 | 6,854 (27.6) | | 503 (30.0) | 0.034 |
| Previous Atrial fibrillation | 20,802 (8.3) | 5,893 (2.7) | | <0.001 | 16,124 (29.1) | 1,823 (18.2) | <0.001 | 9,419 (37.9) | | 479 (28.6) | <0.001 |
| Previous Diabetes | 31,443 (12.5) | 17,809 (8.0) | | <0.001 | 16,932 (30.5) | 3,579 (35.6) | <0.001 | 8,282 (33.4) | | 777 (46.4) | <0.001 |
| Previous chronic obstructive pulmonary disease (COPD) | 18,382 (7.3) | 6,480 (2.9) | | <0.001 | 12,199 (22.0) | 1,774 (17.7) | <0.001 | 6,047 (24.4) | | 386 (23.0) | 0.238 |
| Previous transient ischaemic attack (TIA)/stroke | 4,476 (1.8) | 1,883 (0.8) | | <0.001 | 6,420 (11.6) | 1,196 (11.9) | 0.335 | 5,905 (23.8) | | 436 (26.0) | 0.040 |
| Previous coronary artery bypass graft (CABG)/ percutaneous coronary intervention (PCI) | 5,078 (2.0) | 3,324 (1.5) | | <0.001 | 1,048 (1.9) | 360 (3.6) | <0.001 | 284 (1.1) | | 46 (2.7) | <0.001 |
| Previous Pulmonary hypertension | 1,061 (0.4) | 233 (0.1) | | <0.001 | 1,173 (2.1) | 104 (1.0) | <0.001 | 705 (2.8) | | 28 (1.7) | 0.006 |

**Table S3.** Comparision of demographic statistics by quintile of revascularisation rate by postcode (instrumental variable) for low risk frailty patients.

|  | Quintile 1 | Quintile 2 | Quintile 3 | Quintile 4 | Quintile 5 |
| --- | --- | --- | --- | --- | --- |
| Total | 100,376 | 104,035 | 99,103 | 93,158 | 76,478 |
| Sex (male) | 61.0% | 63.2% | 64.1% | 64.9% | 66.0% |
| Ethnicity |  |  |  |  |  |
| White | 92.6% | 89.8% | 89.6% | 90.8% | 89.6% |
| Black | 1.4% | 1.8% | 1.4% | 0.9% | 1.1% |
| Asian | 4.4% | 6.4% | 7.2% | 6.9% | 8.0% |
| Other/Mixed | 1.5% | 2.1% | 1.8% | 1.4% | 1.3% |
| Age | 69.7 (14.1) | 69.1 (14.1) | 69.0 (13.4) | 68.9 (13.9) | 68.4 (13.8) |
| Deprivation quintile |  |  |  |  |  |
| Least deprived | 15.5% | 18.2% | 17.1% | 19.8% | 18.4% |
| 2 | 19.8% | 18.7% | 20.1% | 20.8% | 19.5% |
| 3 | 21.5% | 20.3% | 21.5% | 19.6% | 20.2% |
| 4 | 20.3% | 20.9% | 21.2% | 20.7% | 19.1% |
| Most deprived | 23.0% | 21.9% | 20.0% | 19.1% | 22.8% |

**Table S4.** Comparision of demographic statistics by quintile of revascularisation rate by postcode (instrumental variable) for intermediate risk frailty patients.

|  | Quintile 1 | Quintile 2 | Quintile 3 | Quintile 4 | Quintile 5 |
| --- | --- | --- | --- | --- | --- |
| Total | 15,780 | 14,907 | 13,505 | 11,975 | 9,328 |
| Sex (male) | 47.7% | 49.4% | 49.3% | 50.6% | 50.9% |
| Ethnicity |  |  |  |  |  |
| White | 95.0% | 92.0% | 91.8% | 92.3% | 91.3% |
| Black | 1.3% | 1.8% | 1.5% | 1.1% | 1.2% |
| Asian | 2.9% | 5.0% | 5.6% | 5.6% | 6.7% |
| Other/Mixed | 0.9% | 1.2% | 1.0% | 0.9% | 0.8% |
| Age | 78.8 (11.7) | 78.6 (11.8) | 78.7 (11.6) | 78.6 (11.7) | 78.0 (11.9) |
| Deprivation quintile |  |  |  |  |  |
| Least deprived | 13.9% | 16.6% | 15.4% | 17.8% | 15.7% |
| 2 | 18.3% | 16.8% | 19.7% | 18.9% | 18.1% |
| 3 | 21.6% | 21.4% | 22.6% | 20.6% | 20.9% |
| 4 | 21.6% | 22.2% | 21.7% | 21.9% | 21.0% |
| Most deprived | 24.6% | 23.1% | 20.6% | 20.7% | 24.4% |

**Table S5.** Comparision of demographic statistics by quintile of revascularisation rate by postcode (instrumental variable) for high risk frailty patients.

|  | Quintile 1 | Quintile 2 | Quintile 3 | Quintile 4 | Quintile 5 |
| --- | --- | --- | --- | --- | --- |
| Total | 6,649 | 6,283 | 5,483 | 4,658 | 3,424 |
| Sex (male) | 42.0% | 43.3% | 43.3% | 43.2% | 44.5% |
| Ethnicity |  |  |  |  |  |
| White | 95.9% | 93.4% | 93.3% | 94.0% | 93.0% |
| Black | 1.3% | 2.0% | 1.5% | 1.1% | 1.5% |
| Asian | 1.9% | 3.3% | 4.0% | 4.3% | 4.8% |
| Other/Mixed | 0.9% | 1.3% | 1.2% | 0.6% | 0.8% |
| Age | 81.7 (10.5) | 81.6 (10.4) | 81.8 (10.1) | 81.6 (10.3) | 81.3 (10.5) |
| Deprivation quintile |  |  |  |  |  |
| Least deprived | 13.6% | 16.1% | 14.4% | 16.9% | 15.2% |
| 2 | 18.0% | 17.2% | 18.5% | 19.1% | 17.3% |
| 3 | 22.4% | 20.5% | 22.8% | 21.1% | 21.5% |
| 4 | 20.7% | 22.4% | 23.4% | 22.8% | 22.8% |
| Most deprived | 25.3% | 23.9% | 20.8% | 20.1% | 23.3% |

**Table S6.** Logistic model for investigations and treatments within six months from index date adjusted for patient characteristics and comorbidities by frailty level. MRI rates are not presented due to the small number of patients undergoing MRI.

|  | Odds ratio | P-value |
| --- | --- | --- |
| Angiography |  |  |
| Intermediate frailty | 0.37 (0.36-0.38) | <0.001 |
| High frailty | 0.17 (0.16-0.18) | <0.001 |
| Echo cardiogram |  |  |
| Intermediate frailty | 0.81 (0.79-0.82) | <0.001 |
| High frailty | 0.62 (0.60-0.64) | <0.001 |
| Coronary artery bypass graft (CABG) |  |  |
| Intermediate frailty | 0.35 (0.33-0.37) | <0.001 |
| High frailty | 0.09 (0.98-0.11) | <0.001 |
| Purcutaneous coronary intervention (PCI) |  |  |
| Intermediate frailty | 0.47 (0.45-0.48) | <0.001 |
| High frailty | 0.25 (0.23-0.26) | <0.001 |
| Medical management |  |  |
| Intermediate frailty | 2.47 (2.41-2.54) | <0.001 |
| High frailty | 5.02 (4.76-5.30) | <0.001 |

All models were adjusted for age, sex, prior CAD, prior CHF, prior CKD, prior atrial fibrillation, prior diabetes, prior COPD, prior stroke, prior CABG or PCI, prior pulmonary hypotension, ethnicity, social deprivation and diagnosis. Low-risk frailty was used as the reference level in all models.

**Table S7.** Causes of death as defined by the first letter in the ICD-10 code by frailty level.

| Primary cause of death (ICD-10 group) | Frailty level | | |
| --- | --- | --- | --- |
|  | High N=26,504 | Intermediate N=65,522 | Low N=473,352 |
| Certain infectious and parasitic diseases (A, B) | 272 (1.1%) | 1,939 (1.2%) | 636 (1.0%) |
| Malignant neoplasms (C) | 1,763 (7.0%) | 33,465 (11.3%) | 6,263 (16.3%) |
| Endocrine, nutritional and metabolic diseases (E) | 715 (2.9%) | 3,550 (2.6%) | 1,436 (1.7%) |
| Mental, behavioural and neurodevelopmental disorders (F) | 2,682 (10.7%) | 9,580 (6.6%) | 3,675 (4.7%) |
| Diseases of the nervous system (G) | 1,037 (4.1%) | 4,903 (2.9%) | 1,577 (2.4%) |
| Diseases of the circulatory system (I) | 10,905 (43.6) | 97,923 (47.8) | 24,953 (45.1) |
| Diseases of the respiratory system (J) | 4,314 (17.2%) | 28,668 (17.0%) | 9,421 (14.0%) |
| Diseases of the digestive system (K) | 920 (3.7%) | 7,904 (4.1%) | 2,247 (3.9%) |
| Diseases of the skin and subcutaneous tissue (L) | 95 (3.7%) | 662 (3.9%) | 224 (4.1%) |
| Diseases of the muscoskeletal system and connective tissue (M) | 247 (1.0%) | 1,406 (1.0%) | 540 (0.7%) |
| Diseases of the genitourinary system (N) | 822 (3.3%) | 3,850 (2.7%) | 1,516 (1.9%) |
| Symptoms, signs and abnormal clinical and laboratory findings, not elsewhere classified (R) | 454 (1.8%) | 2,384 (1.5%) | 819 (1.2%) |

Table showing percentage of patients stratified by frailty level with each ICD-10 disease group as their primary cause of death. ICD-10 groups with <1% of deaths in the high frailty group were omitted from the table.

**Table S8.** Competing causes of cardiovascular mortality.

| Pulmonary heart disease (I26-I28) | 3,153 (0.3) |
| --- | --- |
| Hypertensive diseases (I10-I16) | 17,060 (16.4) |
| Other forms of heart disease (I30-I52 (excluding I50 Heart Failure) | 28421 (27.3) |
| Aortic embolism and dissection, other peripheral vascular diseases, arterial embolism and thrombosis and septic arterial embolism (I71, I73, I74 and I76) | 7,335 (7.1) |
| Other unspecified disorders of the circulatory system (I95-I99) | 487 (0.0) |
| Myocardial infarction (I21-I23) | 97,653 (94.1) |
| Stroke (I60-I66 and I69) | 21,464 (20.7) |
| Heart failure (I50) | 73,962 (71.3) |

**Table S9.** All-cause and Cardiovascular mortality based on gender and revascularisation.

|  | Female | | Male | |
| --- | --- | --- | --- | --- |
|  | Revascularisation  (n=745) | No revascularisation  (n=14,335) | Revascularisation  (n=930) | No revascularisation  (n=10,494) |
| One year cardiovascular mortality | 221 (29.7%) | 6,352 (44.3%) | 250 (26.9%) | 4,956 (47.2%) |
| One year all-cause mortality | 235 (31.5%) | 7,890 (45.0%) | 289 (31.1%) | 6,215 (59.2%) |

**Table S10.** Five-year survival probability comparing patients who received revascularisation with those who did not, stratified by frailty level. Due to missing data models were fitted on 553,767 patients.

| Frailty level | No revascularisation | Revascularisation |
| --- | --- | --- |
| Low | 0.61 (0.61-0.61) | 0.77 (0.77-0.78) |
| Intermediate | 0.22 (0.22-0.23) | 0.45 (0.44-0.46) |
| High | 0.11 (0.11-0.11) | 0.26 (0.25-0.28) |

**Additional instrumental variables methodology**

Confounders can cause bias when variables are correlated with the explanatory variable of interest and the outcome in a statistical model. Where information on confounding variables is available within a dataset, these can be included in the model to adjust for this bias. However, when unknown variables or unobserved correlations of these variables are not included in the dataset, techniques such as instrumental variables can be used. An effective instrumental variable is exogenous and is correlated to the explanatory variable but only affects the outcome variable through the explanatory variable rather than directly.

For this study, the explanatory variable of interest was whether a patient received revascularisation, and the primary outcome variable was mortality. Measured confounders were included in the models, but there were concerns that, although the patients were stratified by frailty, there would likely be differences in the severity of symptoms for ACS patients that would influence both the likelihood of revascularisation and mortality risk. These health factors can be assessed clinically, but one of the limitations of the HES dataset is that these observations, such as the GRACE score, are unavailable. The quintile of revascularisation rate by outward postcode was selected as a potentially suitable instrumental variable as it would be expected to affect patients' likelihood of revascularisation. Still, the postcode-related variation would only be expected to influence mortality through the explanatory variable of whether a patient received revascularisation. Instrumental variable techniques are generally developed for linear modelling outcomes, but one/five-year mortality is a binary outcome. Recursive Bivariate Probit models (RBIProbit, STATA), a Stata package that uses an instrumental variable methodology for binary outcomes, was used for these analyses. (20)

A statistical test to evaluate the correlation of the instrumental variable and the explanatory variable was carried out by comparing the first stage F-statistic with the Stock-Yogo critical value to determine the strength of the quintile of revascularisation rate by outward postcode as an instrumental variable. To check that the instrumental variable was not linked to the unrecorded confounders, demographic characteristics of patients in each quintile of the instrumental variable were created and stratified by frailty level, where a trend over quintiles in measured confounders could indicate a potential trend in unmeasured confounders.

**Statements (RECORD Checklist)**

Validation of data (RECORD 6.2): The codes used to identify diagnosis, treatments and outcomes were selected by a cardiac surgeon. Numbers of STEMI, NSTEMI and unstable angina diagnoses were counted per year, and trends in two ICD-10 codes, where it was unclear which diagnosis they indicated, were also tracked per year to ensure that we were using the correct codes to identify the correct numbers of each diagnosis that would be expected in each year. Cardiac surgeons provided information on how many patients they would expect to receive interventions and treatments based on their clinical expertise, and we checked our findings against this information.

Numbers of patients with linked data (RECORD 6.3): The HES APC dataset was linked with ONS mortality data. This linkage was carried out by NHS Digital using their linkage methodology. Any ONS records that cannot be successfully matched using this methodology are not provided in the ONS mortality dataset. This means there is no loss of participants when linking the data and all patients without ONS data are assumed to be alive at the end of follow-up.

Data access and cleaning methods (RECORD 12.1): Authors JM and FL had full access to the datasets used in this research. JM performed the data cleaning and analysis, and FL supervised the cleaning and analysis for this project.

Linkage (RECORD 12.3)**:** All datasets were provided by NHS Digital. NHS Digital carried out all data linkage, and an anonymised ID was given to each patient to enable researchers to link their data across datasets.

Accessibility of protocol, raw data, and programming code (RECORD 22.1): Linked HES and ONS mortality data would be accessed through an agreement with NHS Digital. Statistical code is available upon request from jm@leicester.ac.uk.
